# Successes and challenges of implementing a cancer care delivery intervention in community oncology practices: lessons learned from SWOG S1415CD

**DOI:** 10.1186/s12913-022-07835-4

**Published:** 2022-04-01

**Authors:** Kate K. Watabayashi, Ari Bell-Brown, Karma Kreizenbeck, Kathryn Egan, Gary H. Lyman, Dawn L. Hershman, Kathryn B. Arnold, Aasthaa Bansal, William E. Barlow, Sean D. Sullivan, Scott D. Ramsey

**Affiliations:** 1 Fred Hutchinson Cancer Research Center, 1100 Fairview Ave N. PO Box 19024, Seattle, WA 98109 USA; 2 Amazon, 410 Terry Ave N., Seattle, WA 98109 USA; 3 School of Medicine, University of Washington, Seattle Cancer Care Alliance, 825 Eastlake Ave East, Seattle, WA 98109 USA; 4 Columbia University Medical Center, 161 Ft. Washington 1068, New York, NY 10032 USA; 5 SWOG Statistics and Data Management Center, 1100 Fairview Ave N., Seattle, WA 98109 USA; 6 CHOICE Institute, School of Pharmacy, University of Washington, University of Washington Health Sciences Building, 1956 NE Pacific St. H362, Seattle, WA 98195 USA

**Keywords:** Cancer care delivery, Intervention, Colony stimulating factor, Cluster-randomized, Computerized clinical decision support system

## Abstract

**Background:**

Cancer Care Delivery (CCD) research studies often require practice-level interventions that pose challenges in the clinical trial setting. The SWOG Cancer Research Network (SWOG) conducted S1415CD, one of the first pragmatic cluster-randomized CCD trials to be implemented through the National Cancer Institute (NCI) Community Oncology Program (NCORP), to compare outcomes of primary prophylactic colony stimulating factor (PP-CSF) use for an intervention of automated PP-CSF standing orders to usual care. The introduction of new methods for study implementation created challenges and opportunities for learning that can inform the design and approach of future CCD interventions.

**Methods:**

The order entry system intervention was administered at the site level; sites were affiliated NCORP practices that shared the same chemotherapy order system. 32 sites without existing guideline-based PP-CSF standing orders were randomized to the intervention (*n* = 24) or to usual care (*n* = 8). Sites assigned to the intervention participated in tailored training, phone calls and onboarding activities administered by research team staff and were provided with additional funding and external IT support to help them make protocol required changes to their order entry systems.

**Results:**

The average length of time for intervention sites to complete reconfiguration of their order sets following randomization was 7.2 months. 14 of 24 of intervention sites met their individual patient recruitment target of 99 patients enrolled per site.

**Conclusions:**

In this paper we share seven recommendations based on lessons learned from implementation of the S1415CD intervention at NCORP community oncology practices representing diverse geographies and patient populations across the U. S. It is our hope these recommendations can be used to guide future implementation of CCD interventions in both research and community settings.

**Trial Registration:**

NCT02728596, registered April 5, 2016.

**Supplementary Information:**

The online version contains supplementary material available at 10.1186/s12913-022-07835-4.

## Background

Cancer Care Delivery (CCD) research studies evaluate changes to the way clinics deliver treatment and services to improve patient outcomes [1]. In 2014, the National Cancer Institute (NCI), recognized the importance of CCD research to generate evidence-based practice change by allocating funding through the NCI Community Oncology Research Program [2] (NCORP), a national network bringing cancer clinical trials and care delivery studies to community settings. Despite increased support for CCD research, studies evaluating alterations in clinic workflow or processes remain relatively uncommon. Lessons learned from studies evaluating practice-level changes are therefore useful, both as roadmaps for the clinical uptake of evidence-based interventions and as learnings for improvements in future study design and implementation.

TrACER (Trial Assessing CSF Prescribing Effectiveness and Risk) [3] is one of the first pragmatic cluster-randomized CCD trials to be implemented through the NCORP program. The study is designed to compare a guideline-informed intervention of primary prophylactic colony stimulating factor (PP-CSF) standing orders compared to usual care in community practice. Oncology practice guidelines recommend PP-CSF for chemotherapy regimens carrying a high risk (> 20%) of febrile neutropenia (FN), and to consider using PP-CSF for intermediate risk regimens (10%-20%).

Many studies across healthcare settings have tested the use of modifications to electronic health records (EHR) to improve care through standing orders for prescriptions and standardized protocols [4]. However, little literature exists on how to successfully implement such interventions in community oncology practices. TrACER aimed to better align PP-CSF prescribing practices in the community with clinical evidence and improve knowledge of the effectiveness of PP-CSF in intermediate risk chemotherapy regimens. The intervention required practice-level modifications to chemotherapy ordering systems to include guideline-based PP-CSF standing orders. The intervention approach was modeled on a regional pilot study [5] to test methods to improve adherence to American Society of Clinical Oncology (ASCO) Choosing Wisely [6] recommendations in Washington State and was scaled up and adapted for use in the cluster-randomized clinical trial setting.

In this paper we share challenges and lessons learned from the implementation of the intervention in this large-scale cluster-randomized CCD study. The intervention for TrACER provides an exemplar of the CCD intervention paradigm, including considerations of clinical engagement and maintaining fidelity across multiple EHR platforms.

## Methods

### Setting

TrACER (NCT02728596) was financially supported by the Patient-Centered Outcomes Research Institute’s (PCORI) Large Pragmatic Trials initiative and by the NCORP, a national network comprised of approximately 1,000 community oncology practices that are organized into “sites.” Fourteen NCORP designated sites are categorized as minority/underserved (MU-NCORP) sites that document patient populations comprised of at least 30% racial/ethnic minorities or rural residents [7]. The unit of randomization for TrACER is a single or group of NCORP practices that share a common chemotherapy order entry system. In this paper we refer to the unit of randomization as a “site” and the NCORP network of community-based practices as “NCORP practices.” Recruitment efforts, regulatory compliance and data collection were managed by the SWOG Cancer Research Network (SWOG) [8]. SWOG is a global cancer research community that designs and conducts publicly funded clinical trials and is part of the NCI National Clinical Trials Network [9].

### Study design

TrACER is a pragmatic study with a cluster-randomized design. In cluster designs, the intervention is administered at the group or clinic level rather than the individual level. Eligible NCORP practices were required to have NCORP CCD designation and treat at least 60 breast, non-small cell lung or colorectal cancer adult patients annually with chemotherapy. Details of the design are described in a separate publication [3]. Sites were randomly assigned to the intervention or usual care arms using a 3:1 randomization scheme and stratified by site size (patient volume at the site) and MU-NCORP status (designated MU-NCORP vs. non-MU-NCORP). Intervention sites were required to add the study standing orders to all corresponding order templates to ensure adequate capture of patients receiving chemotherapies of varying FN risk levels.

Patient eligibility criteria are intentionally broad for pragmatic studies to better represent the population and care observed in real-life community practice settings. Patients were eligible if they were at least 18 years old, diagnosed with breast, non-small cell lung (NSCLC), or colorectal cancer and prescribed a protocol-allowed chemotherapy regimen. To ensure the study was adequately powered to detect outcomes for the sub-randomization, all sites were assigned the same patient recruitment target of 99 patients per site. Sites could exceed their target up to a maximum cap of 200 patients per site. All patient activities took place after approval by a local institutional review board (IRB) and in accordance with an assurance filed with and approved by the U.S. Department of Health and Human Services.

To ensure our study design was feasible and highly relevant to community oncology practices, we convened a 21-member external stakeholder advisory group (ESAG) to work closely with the study team during all phases of the project. ESAG members included patient advocates, payers, pharmacists, national guidelines experts, providers, and a medical ethicist [10]. ESAG subject matter experts contributed directly to implementation of the intervention by providing input on the design of materials to assess study site readiness and participated in annual reviews of the list of protocol-allowed chemotherapy regimens.

### Site recruitment

To maximize recruitment efficiency, low-volume sites (< 60 breast, non-small cell lung and colorectal cancer patients seen per year) were excluded. Potentially eligible NCORP practices were targeted with group emails via a NCORP CCD research distribution list, and the study was advertised during in-person NCI meetings for the cooperative groups NRG Oncology [11], Alliance for Clinical Trials in Oncology [12], The ECOG-ACRIN Cancer Research Group [13], and SWOG. Research staff at interested practices were required to submit a site application used to determine site eligibility and assess the feasibility of conducting the study at each site. The application included questions about the type of existing order system at the site (EHR, paper), the feasibility of completing the changes within 6 months from randomization, and estimated length of time for local IRB review. Completed applications were reviewed by the research team and eligible sites were invited to participate.

### Intervention: ordering system modifications

TrACER has four study arms: three randomized arms (two intervention, one usual care) and one non-randomized observational cohort arm. Sites that already had automated standing orders for PP-CSF prescribing were assigned to a non-randomized observational cohort (Arm 1). The remaining sites were randomized to usual care (Arm 2) or intervention. All sites randomized to the intervention modified their order systems to exclude PP-CSF for low-risk regimens and include PP-CSF for high-risk regimens. Intervention sites underwent a secondary randomization where they were assigned to either include (Arm 3) or exclude (Arm 4) PP-CSF automated standing orders for chemotherapy regimens classified as intermediate risk for FN. In this paper we focus on the implementation of the intervention at sites randomized to study Arms 3 and 4.

**Fig. 1:**
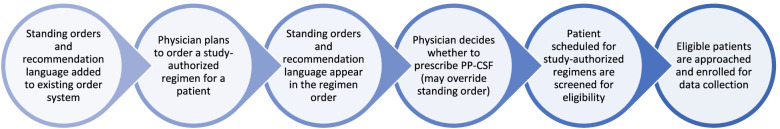
Intervention to patient enrollment workflow

The intervention required study sites to reconfigure their existing chemotherapy order sets to include or exclude PP-CSF standing orders in accordance with the secondary randomization assignment. The exact format of the order sets depended upon site infrastructure and capacity, encompassing EHR-based order entry systems, pre-printed paper templates, and process workflows. Intervention system modifications were embedded into the chemotherapy regimen order set prior to the selection of the chemotherapy treatment and patient enrollment (Fig. 1).

### Implementation

**Fig. 2:**
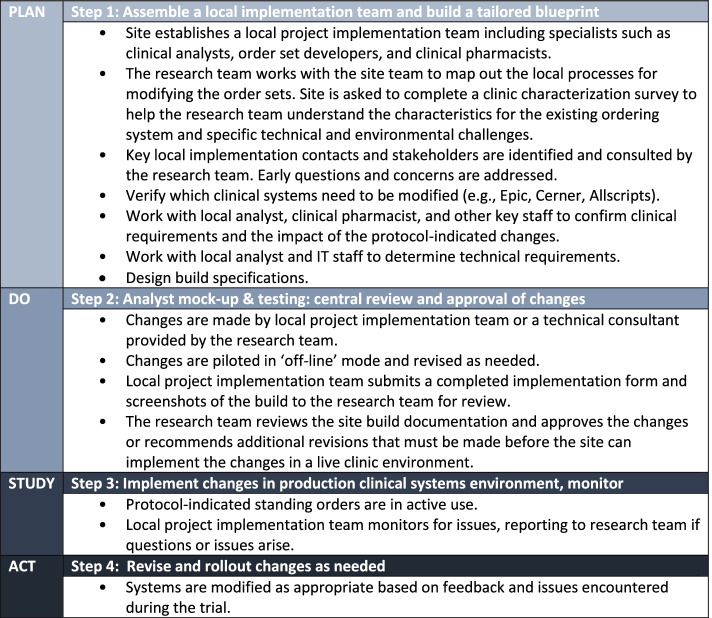
Process to establish a primary prophylactic colony stimulating factor entry protocol

Project staff engaged with professionals at each of the intervention sites involved in technical support or chemotherapy prescribing such as EHR specialists, oncologists, pharmacists, and nurses. EHR consultants were hired to assist as needed with system modifications and troubleshooting. After randomization, intervention sites were provided with a detailed protocol describing four phases of the implementation process with step-by-step instructions for what to expect during each phase (Fig. 2). These steps were modeled on the Plan-Do-Study-Act (PDSA) cycle, a four-stage model for continuous quality improvement management that has been used to implement cancer care delivery quality improvement interventions [14–18]. Clinical site staff were given access to a training video that provided a high-level overview of the reconfiguration process. This was followed by two interactive webinars hosted by the project team providing an overview of the implementation process. After the webinars, we compiled and published a TrACER FAQ on the SWOG TrACER protocol webpage that was regularly updated by project staff.

To support intervention sites through the process of modifying their order systems we held individual calls to discuss the expectations of the intervention with each site, get to know the local implementation team and answer questions. EHR consultants participated in these calls for sites that used the most commonly encountered EHR systems in the study: Epic/Beacon, Aria, Intellidose, Mosaiq and Cerner. We also hosted several in-person TrACER meetings where investigators and project staff presented detailed information about the intervention workflow and demonstrated how modified order sets would perform across different platforms. Reimbursement of $5,000 pre-implementation and $1,500 post-implementation was provided to sites to defray costs of EHR reconfiguration. The project team maintained close contact with sites leading up to, during and after the intervention implementation phase, primarily by phone and email.

During the patient enrollment phase, the study team issued five revisions to the list of protocol-allowed chemotherapy regimens in response to national guideline updates and site requests to add more commonly used regimens. Technical consultant assistance was offered to sites if needed to support modifications made to the standing orders after the intervention was implemented.

## Results

Recruitment of NCORP practices opened in January 2016 and closed in December 2016. Forty-five study sites were enrolled and 24 were randomized to the intervention (12 each in Arm 3 and Arm 4). Reasons for site decline were varied and included a lack of research staff capacity, unwillingness to modify the order system as required for the intervention, site was scheduled to switch to a new EHR system during the trial, and institutional changes such as company re-organizations. Intervention sites represented 17 U.S. states and territories in urban, suburban, and rural locations (Additional file 1). 3,665 total patients were enrolled in TrACER between September 2016 and April 2020. 2,287 patients were enrolled at intervention sites, 1,296 in Arm 3 and 991 in Arm 4. Overall, 53% of randomized sites met their individual accrual targets (Table 1).

**Table 1:** Site order set reconfiguration and enrollment details

	**Intervention Arm 3**	**Intervention Arm 4**	**Intervention combined arms**	**Usual Care**
Number of sites	12	12	24	8
Minority/Underserved sites^a^	5	5	10	4
Average oncology clinics per site				
Average	4	2	6	4
Range	(1, 12)	(1, 9)	(1, 12)	(1, 12)
Months to first accrual from randomization				
Average	8.7	8.2	8.5	7.5
Range	(4, 15)	(3, 12)	(3, 15)	(4,17)
Average accruals per site	108	82.6	95.3	82.4
Met individual site recruitment target	8	6	14	3
Chemotherapy order system				
EHR	8	11	19	N/A
Paper	4	1	5	
EHR consultant available	8	10	18	N/A
Months to complete order set changes				
Average	7.3	7.0	7.2	N/A
Range	(4, 12)	(2, 11)	(2, 12)	

^a^stratification factor

Table 1 compares the length of time to complete the intervention order set configuration and enroll patients for the 32 randomized sites. Only the sites participating in the intervention (*n* = 24) were required to modify their order sets to include the protocol directed standing orders and system alerts. EHR consultants were available for the most widely encountered systems among intervention sites: Epic/Beacon, Intellidose, Mosaiq, Aria, and Cerner.

Order set reconfiguration across all intervention sites took 7.2 months on average per site (range 2–12) (Table 1). Six different EHR ordering systems were represented among intervention practice groups, and five groups used paper ordering (Table 2). Several practice groups utilized multiple platforms within one health system, where one type of platform is used for chemotherapy, and another for supportive medications such as CSFs. Some used a hybrid of EHR and paper ordering. The type of EHR platform did not make much difference to the length of time required for reconfiguration, however paper-based sites were on average slower to complete the changes than EHR sites (10.2 months vs. 6.4 months, respectively) (Table 2). All intervention sites successfully completed reconfiguration of their order sets and opened to patient enrollment within 15 months from randomization.

**Table 2:** Time to complete reconfiguration, by type of order system

	**Intervention Sites (** ***N*** ** = 24)**	**Average months to complete reconfiguration (range)**
EHR system, all	19	6.4 (2, 12)
Aria	3	6.3 (2, 10)
Cerner	2	9.5 (7, 12)
Eclipsys	1	3.0 (n/a)
Epic/Beacon	9	6.4 (2, 7)
Intellidose	2	5.5 (5, 6)
Mosaiq	2	5.5 (4, 7)
Paper system	5	10.2 (8, 12)

## Discussion

TrACER’s practice-level standing order intervention presented our team with unique operational opportunities that could have implications for the design and implementation of future CCD interventions. The Practical, Robust, Implementation and Sustainability Model (PRISM) is a commonly used tool to help translate research findings into practice by highlighting activities associated with the success of intervention implementation and sustainability [15]. Although PRISM is not intended to guide the implementation of interventions within a clinical trial, the tool is highly relevant to implementing research in a pragmatic setting and provides a useful framework for sharing our findings. Here we use the PRISM domains to help distill our experience implementing TrACER into seven recommendations broadly applicable to CCD interventions conducted in a research or community setting (Table 3).

**Table 3:** TrACER implementation recommendations mapped to corresponding PRISM domains

PRISM Domain	PRISM key suggestions	TrACER implementation experience
Intervention Organizational Perspective	Assure processes coordinate needs of all stakeholders	Working with clinical stakeholders to identify the most commonly prescribed chemotherapy regimens could have reduced the time and effort needed for sites to implement the reconfiguration. (**recommendation 1**)
Intervention Organizational Perspective	Simplify the intervention while maintaining essential elements	Distilling the intervention requirements into a concise set of expectations interpretable across practice settings increased site buy-in while maintaining fidelity. (**recommendation 2**)
Intervention Organizational Perspective	Assess the usability and adaptability of the intervention	Building flexibility into the protocol and allowing for additional time to implement allowed us to accommodate variation in workflow. (**recommendation 3**)
Intervention Organizational Perspective	Design monitoring so results can be seen early	Collecting feedback during and after the intervention from site principal investigators and clinical staff could have improved delivery of the intervention and informed the design of future CCD trials. (**recommendation 4**)
Characteristics of Organizational Recipients	Use existing staff during early stages to ease implementation	Sites preferred to use their existing IT teams to make changes to their order system so the external technical consultants were used in an advisory and supportive role. (**recommendation 5**)
Characteristics of Organizational Recipients	Work with all levels of management to earn and communicate program support Engage clinical leaders from planning through implementation and maintenance stages	Funding for two full time research support positions allowed the study team to cultivate and sustain strong relationships with clinical leaders, managers and other key staff positions at participating sites, staff multiple channels for communication, and be responsive to site concerns. (**recommendation 6**)
External Environment	Work with policy and decision makers to alleviate burden or provide incentives when possible	We worked with payer stakeholders to provide information about reimbursement for guideline-informed PP-CSF prescribing to help alleviate site concerns and offered to work with them to develop a reimbursement mechanism if this became an issue during the trial. (**recommendation 7**)

### Recommendation #1 During the study design stage, identify community practice patterns that inform the scope and amount of effort required for practice-level changes

Our protocol-approved chemotherapy regimen list included 77 National Comprehensive Cancer Network recognized core regimens and 40 biologic variants for breast, non-small cell lung, and colorectal cancer, meant to capture a broad population and variety of practices and settings. However, the number of regimens hampered institutional buy-in and resulted in a lengthy review process at some sites that delayed study activation by several months. Following activation, sites that struggled to enroll patients cited the exclusion in the protocol of commonly used regimens at their practice as a recruitment barrier. This led us to solicit commonly used regimens directly from the sites, which in turn necessitated frequent updates to the protocol, databases, and site ordering systems. A review conducted two and a half years after study activation of the number of patients enrolled on each regimen found 36 out of the 111 original regimens had no enrollment at all. The 20 highest-accruing regimens accounted for 90% of enrollment. Full accrual was thus achievable with just a few of the most widely used regimens that could have been identified up front by clinical site staff. Pilot testing at a few sites, surveying sites during protocol development and/or involving more clinical staff on our external stakeholder advisory group to help design the intervention would likely have generated a shorter and more relevant list of regimens, reducing the length of time needed for implementation. In addition, soliciting information from clinical site staff on their utilization of regimens through the course of the study could have informed relevant updates to the regimen list to reflect the evolving community practice patterns.

### Recommendation #2 Distill the intervention requirements into a concise set of expectations interpretable across practice settings

Seven unique systems for ordering chemotherapy regimens were used across the intervention sites, including electronic systems, templated paper systems, and non-templated paper systems (Table 2) To accommodate this wide range of modes, we distilled the scope of the intervention into four guiding principles: 1. Recommendation language should be added to the system before the order for PP-CSF; 2. The intervention should impact all study-authorized orders; 3. The intervention should impact all patients on study-authorized orders; and 4. Chemotherapy and PP-CSF ordering should occur before the patient is enrolled in the trial. Developing a clear and concise set of expectations, specific enough to be meaningful yet broad enough to apply to all site workflows, helped maintain the integrity of the intervention across systems. For example, these principles allowed the required passive alert language to be customized to the individual needs and preferences of sites, facilitating buy-in and the adoption of changes into the existing workflow. Allowing flexibility for process and system variation enabled us to accommodate any type of order system we encountered, increasing the potential likelihood of broad clinic uptake in the event of positive findings. We acknowledge the target of our intervention, chemotherapy order systems, worked well with this approach because each site had some type of existing workflow for performing this function. Interventions focused on creating entirely new processes or adopting new technologies may require a different approach.

### Recommendation #3 Build flexibility into the protocol and allow additional time for implementation to accommodate variation in workflow and procedures to track intervention fidelity

TrACER was conceived as an intervention targeting electronic ordering systems, yet an unexpected finding was paper-based systems were the second most common ordering platform at 5 (21%) intervention sites (Table 1). These sites had unique workflows for placing orders requiring individualized adaptations to fit with the principles of implementation. Implementing an EHR-centric intervention in paper ordering systems required more time and effort to develop the workplan and communicate closely with the clinic. To verify changes were implemented correctly, we added in-person site visits from the study team. These visits required extra resources and time but had the benefit of boosting morale and buy-in for paper-based sites who initially had concerns about their ability to implement the intervention. Of note, one third of the MU-NCORP sites participating in TrACER used paper-based ordering. A pragmatic approach to implementation allowed us to adapt our process to accommodate their workflows, contributing to the generalizability of trial results. We also allowed a window of 12 months for sites to complete the order system modifications. This proved necessary because while the majority of sites implemented the intervention within seven months, 29% took 10 months or longer (Table 1). Sites were able to activate at their own pace once their order changes had been validated and approved by the study team.

Although we verified standing orders were set up correctly at the start of the study, we did not have a built-in mechanism for continuous monitoring to confirm orders remained in place or were updated based on protocol revisions. This led to additional work at the end of the trial to audit sites for this information. We recommend future CCD studies include a plan in their protocol for regular monitoring of intervention performance via chart review, informational interviews with sites or some other process appropriate to the study design to proactively track intervention fidelity.

### Recommendation #4 Collect feedback from site principal investigators and clinical staff to inform future CCD research

A limitation of our approach is we did not collect data about sites’ experience of implementing the intervention, e.g., what worked and what didn’t work and how they felt about the modified order sets, to inform future directions. Such information could be useful to guide the design of new cluster-randomized interventions, to refine the implementation approach, or in the context of negative findings to understand why an intervention may not have worked as expected. We recommend studies include processes to gather continuous feedback from participating practices as well as informational interviews or surveys at the end of the study period with clinical staff who were heavily involved with implementation to collect data about the acceptability and feasibility of CCD interventions.

### Recommendation #5 Provide readily accessible and continuous technical support for complex interventions

EHR consultants were hired by the project to assist randomized intervention sites with technical aspects of changing the ordering systems. Consultants engaged early in the implementation process to help the sites understand how to make changes to their systems and create a change workflow. The availability of consultants on the project team provided reassurance to sites who had concerns about the scope of the required changes. During intervention site calls, consultants provided examples of suggested workflows based on the existing EHR platform and helped troubleshoot in real time with local implementation teams. They also assisted with the validation of changes to the order sets prior to study activation. Due to budget constraints, consultants were only available for the most commonly used EHR platforms, covering 75% of sites as shown in Table 1.

### Recommendation #6 Provide sufficient effort for the research team to engage often with sites before, during and after implementation

PCORI funded two full time positions for a research coordinator and a research manager on TrACER. This level of staffing allowed for time to cultivate and sustain strong relationships with the sites. Investment in site engagement facilitated the development of site champions who were instrumental in gaining and maintaining continued institutional engagement for the duration of the trial.

Multiple channels were available for sites to ask questions and provide feedback including kick-off calls with each site, in-person trainings, and a dedicated study email address that was monitored full-time. Having a research coordinator staff these channels allowed the team to be highly responsive to site concerns. For example, the project team adjusted the protocol to include requested chemotherapy regimens and collaborated with external stakeholder advisory group members to create a document with key talking points to assist clinic providers and staff during patient approach.

Having sufficient dedicated effort for these roles allowed for pro-active engagement with the sites and the availability to trouble-shoot issues as they arose. This proved critical to meeting recruitment targets and timelines. Usual care sites, which did not receive the same level of continuous engagement during study start up, were less responsive to outreach from the research team after activation and fewer met their individual recruitment targets (38%) compared with the intervention sites (58%) (Table 1).

### Recommendation #7 Work with clinical and other relevant stakeholders to identify and develop plans to alleviate or address potential barriers to implementation

When we first advertised the TrACER study to NCORP practices, several expressed concern that insurance might not cover the cost of PP-CSF for some study participants, particularly those receiving intermediate risk chemotherapy regimens. We worked with the payer stakeholders on our ESAG to clarify for practices that the majority of carriers would reimburse for PP-CSF if administered per the study recommendations. We also committed to developing a reimbursement assistance mechanism for the trial if participating sites experienced problems with getting PP-CSF reimbursed by insurance. Finally, the protocol allowed physicians to override standing orders at their discretion. These efforts helped gain buy-in from clinical site leaders for the intervention and facilitated site recruitment.

## Conclusions

Despite the challenges of introducing a CCD standing order intervention to community oncology practices, all 24 intervention sites successfully reconfigured their order sets and opened to patient enrollment. Factors such as the flexibility of the intervention design and the investment in full-time staff to provide continuous high-quality engagement with sites facilitated implementation. The pragmatic nature of the study and the strong relationship and communication between the research team and clinic staff enabled us to respond quickly to site concerns and external changes to guidelines and treatment patterns that impacted implementation. Implementation could have benefitted from the inclusion of processes to track and respond to changing prescribing patterns over time and monitor intervention fidelity. We also benefitted from the NCORP providing built-in infrastructure and funding to support CCD research activities and access to a broad and diverse network of community cancer practices. Our experience demonstrates that implementing a CCD order entry system intervention is feasible in a cooperative group setting and provides a framework for implementation of CCD interventions in both research and community settings.

## Supplementary Information

**Additional file 1.**

## Data Availability

The datasets generated and/or analysed during the current study are not publicly available because the trial results have not yet been published but are available from the corresponding author on reasonable request.

## Abbreviations

CCD: Cancer Care Delivery
PP-CSF: Primary prophylactic colony stimulating factor
NCI: National Cancer Institute
NCORP: NCI Community Oncology Research Program
TrACER: Trial Assessing CSF Prescribing, Effectiveness and Risk
FN: Febrile neutropenia
EHR: Electronic Health Record
ASCO: American Society of Clinical Oncology
PCORI: Patient-Centered Outcomes Research Institute
MU-NCORP: Minority/Underserved NCI Community Oncology Research Program
SWOG: SWOG Cancer Research Network
NSCLC: Non-small cell lung cancer
