# Supplementary figures and images for: Successes and challenges of implementing a cancer care delivery intervention in community oncology practices: lessons learned from SWOG S1415CD
Source: BMC Health Serv Res. 2022 Apr 1;22:432. doi: 10.1186/s12913-022-07835-4 (PMC8973954; doi:10.1186/s12913-022-07835-4)

TrACER Site Map

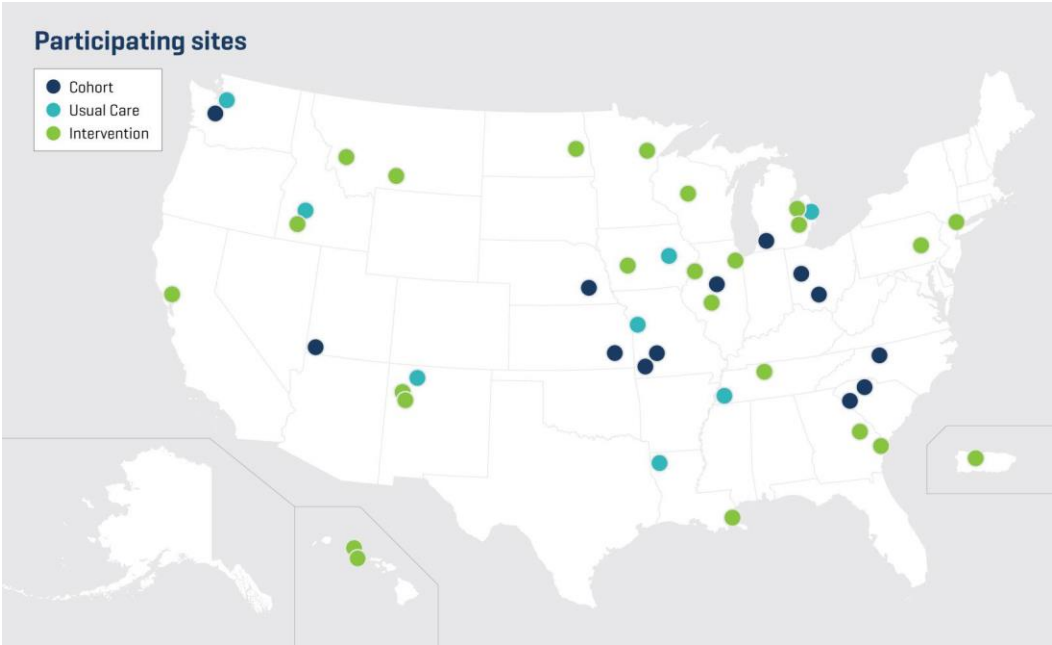

Supplement: Supplementary file 1 — Additional file 1. [file 12913_2022_7835_MOESM1_ESM.pdf]
